# Cultural values and digital gap: Overview of behavioral patterns

**DOI:** 10.1371/journal.pone.0311390

**Published:** 2024-10-01

**Authors:** Maral Jamalova

**Affiliations:** 1 Azerbaijan State University of Economics, Baku, AZ, Azerbaijan; 2 Karabakh University, Khankendi, AZ, Azerbaijan; Tianjin University, CHINA

## Abstract

The study uses different statistical techniques to understand the relationship between variables explaining the digital divide and classification based on The Inglehart-Welzel Cultural Map for 2023. To achieve this purpose variables focusing on Digital Penetration (the percentage of internet and social media users and mobile cellular connections), Operating Systems share (iOS and Android), Device Traffic (laptop/mobile phone-based web traffic) as well as Mobile Commerce variables (bills and payments using mobile internet) were included in the analysis. To minimize any effects arithmetic means of data was calculated.: The results from one-way ANOVA tests indicate significant differences among groups classified by cultural values for almost all measured variables of digitalization. The mean squares and F-values across variables like cellular mobile connections, internet users, and active social media users are significant indicating a shift towards more secular and self-expressive cultural values. The results of the GLM procedure show that significant portions of the total variance in digitalization variables are associated with membership in groups based on the cultural map. This suggests that cultural classifications can explain substantial differences in digital behavior and preferences across populations. Spearman’s correlation coefficients showed strong positive correlations between Traditional/Secular values and several digitalization metrics, such as the use of mobile phones or the internet for payments, and negative correlations with others like share of web traffic by device type (mobile vs. laptop/computer). These correlations suggest that cultural values play a substantial role in influencing digital habits and accessibility.

## Introduction

Digitalization is one of the essential elements of economic and social development [1, 2]. Even though digitalization is linked to demographic indicators such as age, gender, and literacy rate, it is also closely connected with the financial situation [3] and culture [4]. Unequal economic development, differences in religion, beliefs, and values as well as other economic and political factors might cause a big gap between countries. The digital divide refers to the unequal access and utilization of digital technologies and resources among different individuals and communities. It is a term used to describe the disparities in access to and use of digital technologies, such as computers [3], internet connections [5], mobile/smartphones [6] and other digital tools [7].

The digital divide can worsen economic inequalities, as access to digital tools and the internet is essential for enhancing various aspects of an individual’s life. Therefore, analysis of the internet’s evolution and current status focusing on the differences between wired and wireless fixed/mobile internet access are essential aspects for narrowing the digital gap [8]. Broadband Internet access significantly contributes to closing the digital gap by creating economic opportunities, improving education and healthcare, enhancing communication, and providing access to government services and online markets [9]. Expanding broadband access to underserved areas fosters social inclusion and empowers individuals and communities with the tools to thrive in the digital age. Also, bridging the digital gap is essential for creating more equal opportunities for all individuals and communities, and addressing this issue requires a multi-faceted approach involving government initiatives, private sector involvement, and community-based efforts.

The purpose of the study is to define the impact of cultural values (traditional/secular-rational values and survival/self-expression values.) on digitalization-related variables such as the percentage of internet and social media users, share of mobile (iOS and Android) and laptop-based web traffic as well as bills and payments using mobile internet. Therefore, the findings of this study are particularly relevant to the fields designated by JEL codes O33 and O14, as they highlight the complex interplay between technology adoption, the digital gap, and cultural values. The mentioned variables are frequently used in the literature to determine the digital divide [7]. Also, the purpose of the study will help us to understand and address the cultural characteristics of the issues related to the digital divide. Castells [5] illustrates that nowadays civil society movements are closely connected by the Internet and media which are the main communication channels. The study is also designed to determine differences in digitalization-related variables based on the country grouping offered by the World Values Survey Association [10].

## Needs and objectives

The digital divide remains a critical issue that impedes social and economic equality worldwide [11]. This gap in digital access and utilization is influenced by a myriad of factors, including economic conditions [12], infrastructure [8], education levels [13], skills [14] and notably, cultural values [13]. Previous studies in the field illustrated the relationship between digitalization and culture at the local [15] and national level [16, 17]. The findings illustrate that different cultures tend to accept and use technology and services that align with their cultural values [18].

Previous studies mostly used Hofstede’s cultural dimensions [19, 20] to determine the impact of culture on the digitalization [18]. However, the current study has highlighted how cultural dimensions, as defined by the Inglehart-Welzel Cultural Map, impact digital behaviors across different societies. These cultural values—ranging from traditional to secular-rational and from survival to self-expression—correlate strongly with how communities engage with digital technologies. The existence of the relationship, as well as the specific mechanisms through which cultural values influence digitalization-related variables, are not well understood. There remains a critical gap [18] in comprehensive, cross-cultural studies that explore these interactions in depth, particularly how these values affect digital penetration rates, mobile commerce adoption, and device usage patterns. Addressing these gaps is crucial for developing effective interventions aimed at bridging the digital divide in a culturally informed way. Using data from the Inglehart-Welzel Cultural Map to compare with the mean of digitalization-related variables provides new insights into the aforementioned relationship. This approach enables the author to gain a deeper understanding of the prerequisites of the digital divide, aligning with JEL codes O33 and O14. Given the complexities of cultural influences on digital engagement, this study aims to:

1. **Quantify Cultural Impacts**: Systematically assess how traditional/secular and survival/self-expression values influence key digitalization metrics such as internet and social media usage rates, mobile commerce adoption, and the preference for various device types (mobile vs. laptop).
2. **Identify Statistical Differences**: Investigate the presence of statistically significant disparities in digitalization-related data among different cultural groups as classified by the Inglehart-Welzel Cultural Map. This includes exploring how such values might differentially impact digital behaviors in high versus low digital penetration regions.
3. **Measure Variance and Model Cultural Dynamics**: Employ advanced statistical methods to quantify the proportion of variance in digital behaviors explained by cultural classifications. Additionally, model the dynamics of cultural influence to understand potential causal pathways and interactions among cultural values, digital access, and usage patterns.
4. **Develop Cultural Profiles**: Based on the findings, develop detailed profiles of digital engagement strategies tailored to specific cultural contexts. This will involve identifying which aspects of digitalization are most affected by cultural values and proposing targeted interventions that respect and utilize these cultural nuances.

Through these objectives, the research seeks to provide a nuanced understanding of the digital divide, emphasizing the role of cultural values in shaping digital landscapes globally. Insights gained could inform policymakers and practitioners designing culturally adaptive technologies and digital inclusion strategies, thereby enhancing the effectiveness of interventions aimed at closing the digital gap. Therefore, the author is interested in identifying the answers to the above-mentioned research questions:

RQ 1. Are there any statistically significant differences in the means of digitalization-related data grouped based on Inglehart and Welzel’s cultural map?

RQ 2. What is the proportion of the total variance in the mentioned digitalization variables associated with the membership based on Inglehart and Welzel’s cultural map?

RQ 3. What is the strength of the relationship between the mentioned digitalization variables compared to Traditional/Secular (TradAgg) and Survival/Self-Expression (SurvSAgg) values calculated for Inglehart and Welzel’s cultural map?

## Literature review

### Indicators of digital divide

The literature illustrates two different levels of the digital divide [21]. The first level focuses on the fact of having the opportunity, motivation, and ability to access the Internet that briefly might be called ***the accessibility gap*** [5]. As soon as the Internet became more widespread, the authors began to analyze the second level—which is called ***the usage gap*** [22]. The third-level digital divide emphasizes the effects and consequences that arise from access to or the use of digital resources [23, 24]. Considering the rapid development of Artificial Intelligence (AI) and big data, it is important to address digital inequalities, particularly due to their profound influence on various aspects of human life and the global economy [25]. Nowadays, based on the reports of DataReportal, [26] not only the Internet but also smartphones are considered as a tool of accessibility. However, some scholars have already proved that even though the accessibility gap decreases, it does not mean that the usage [27] and consequence-related gaps will also decrease. Therefore, analyzing data regarding the digital divide remains actual.

Broadband internet is crucial for modern society, fostering economic growth and access to information. However, its expansion has widened the digital divide, particularly between urban and rural areas, where infrastructure costs limit access [8, 13, 21]. While mobile broadband, including 4G and 5G, offers a more accessible solution for underserved regions, it cannot fully replace high-speed fixed connections essential for activities requiring higher data rates [9, 21]. The socio-economic impact of broadband is significant, as better access correlates with greater economic participation and social engagement, while inadequate access deepens marginalization [21].

The rapid advancements in Artificial Intelligence (AI), Machine Learning (ML), and cryptocurrencies have significant implications for the digital divide, potentially widening it if not addressed inclusively. While AI [28] and ML [29] offer transformative benefits, they often require advanced digital literacy and skills [30], which are unevenly distributed, leaving those without access or knowledge increasingly marginalized [31]. Similarly, cryptocurrencies have the potential to enhance financial inclusion [23], especially for unbanked populations, but their adoption is hindered by a lack of understanding and infrastructure, particularly in developing regions. Thus, these technologies, while promising, risk exacerbating the digital divide unless efforts are made to improve digital literacy, expand access, and ensure equitable technological advancements [8, 23].

To understand the impact of cultural values on the digital divide, the author selected the most available indicators explaining the phenomena. The number of cellular connections was included as one of the first variables. It is one of the crucial variables as there is a piece of evidence regarding the “smartphone only” group that is more likely to use handsets for access to the Internet [32, 33]. Usually, this group of customers is younger and there is evidence regarding differences in usage patterns.

Recent studies also proved that complex but portable devices such as smartphones can also be one of the indicators illustrating evidence of the digital divide in developing countries [34–36]. It was also previously proved that smartphone adoption is closely connected with GDP, income level as well as operating systems, and other economic variables [12, 37]. Previous studies proved that income level directly influences smartphone choice based on iOS and Android market share and this effect is comparatively higher in developing societies [12]. Moreover, the smartphone market share was not impacted by Gross Savings in 2016 [37]. Therefore, the author of the current study also included a percentage of cellular mobile connections, and prices of smartphones in the analyses. Considering that smartphones have also shaped access to information [22], the percentage of the total population that uses social media became one of the crucial variables [38]. To understand and illustrate the concept of the digital divide author included not only digital penetration-related variables (the Internet user, mobile connections, active social media users) but also variables that determine its impact on the digital divide (Economic Accessibility of Technologies and Mobile Commerce), and some more information regarding making purchases and paying bills via the Internet or mobile phone.

Conceptual definitions for internet skills [14] are used to define the levels of the skills applied for the mentioned operations. Based on the classification mentioned skills were divided into operational (opening, navigating, saving the material), formal (navigating and recognizing different formats), information (locating the information), strategic (goal-oriented actions) [14, 27]. The author of the current work might argue that using mobile phone/internet to pay bills (past year) and making a purchase using mobile phone/internet requires strategic internet skills.

### World value survey

Ronald Inglehart and Christian Welzel proposed the cultural map arguing that there are two main indicators of cross-cultural difference [39]. The findings illustrated on the map are based on the European Value Survey. The World Value Survey, examines freedom, culture, the impact of religion, equality, and other values in more than 110 countries in 2023 [10].

The cultural map [40] combines previously discussed values in two main dimensions named traditional/secular (TradAgg) and survival/self-expression values (SurvSAgg) [39]. Based on the data obtained from the last version of the Cultural map (2023), countries are combined into 8 groups. The countries belonging to the African-Islamic group are comparatively in difficult conditions. They have high levels of traditional and survival values that usually do not result in positive outcomes. This classification was used as the main categorical variable for ANOVA and General Linear Model analyses.

The traditional/secular values (TradAgg) illustrate cross-national differences, highlighting how societies vary in their emphasis on religious beliefs, authority, and cultural norms [39]. The traditional dimension revolves around religion, family, homeland, and parenthood. The countries situated in the lower left corner have a more powerful sense of national identity along with more well-defined societal roles. Societies with high levels of traditional values are opposed to abortion, divorce, or other personal choices (including euthanasia or suicide). The secular dimension prioritizes the opposite values. According to Inglehart and Welzel [39], secular values are associated with the modernization of the national economy (i.e., a transition from agriculture to manufacturing) and a greater degree of individual freedom.

Survival/Self-expression (SurvSAgg) values also illustrate a range of societal priorities, including the level of tolerance for diversity, the importance placed on environmental protection, gender equality, and an individual’s overall sense of well-being [39]. A greater number of survival values (on the left side of the map) indicates that culture emphasizes physical and financial safety. Survival values emphasize economic and physical security. Societies that prioritize survival values tend to exhibit heightened concerns about their immediate well-being and safety. These concerns can stem from experiences of economic hardship, political instability, or threats to physical health. However, self-expression values prioritize individual autonomy and self-realization. Societies that lean towards self-expression values generally enjoy higher levels of economic security and are more politically stable. This stability fosters a culture where individuals feel safe to express themselves, leading to greater acceptance of diversity, innovation, and democratic governance.

## Research methodology

**Fig 1:**
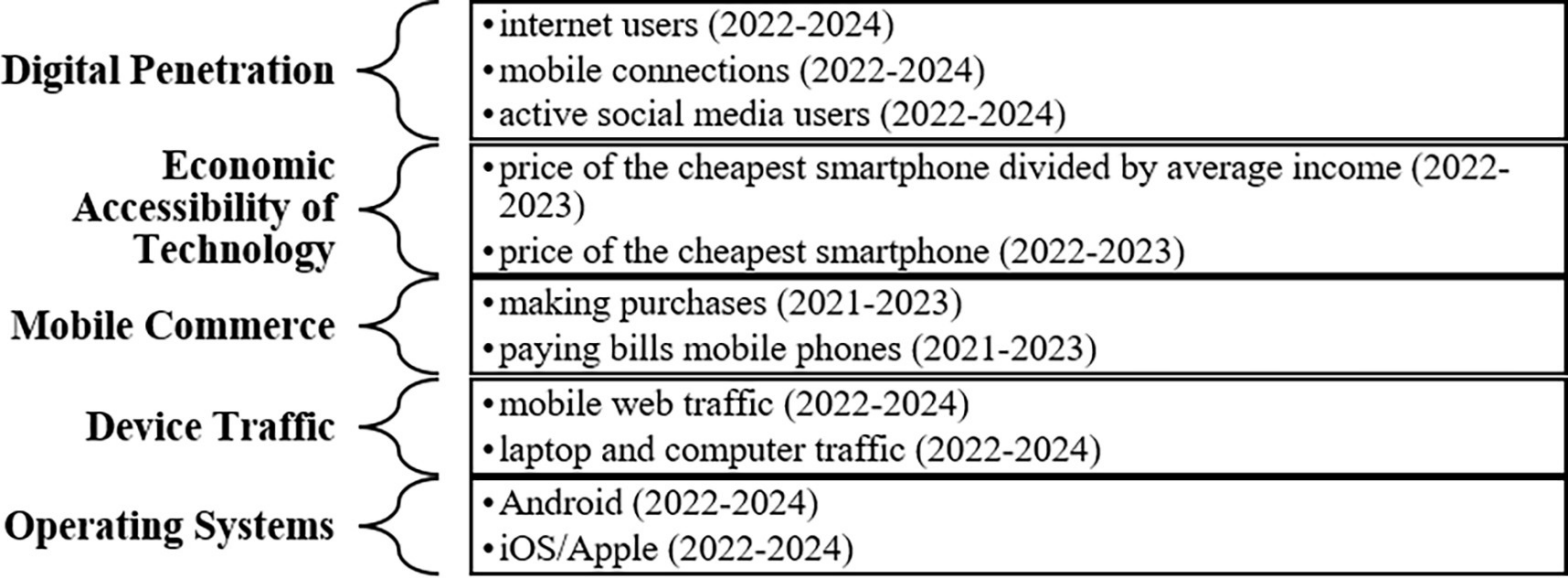
Variables and data collection. Source: Author’s editing.

The author was interested in finding answers to the research questions mentioned in the introductory part of this paper. So, digitalization-related data (i.e., the percentage of internet and social media users, the share of mobile (iOS and Android) and laptop-based web traffic as well as bills and payments using mobile internet) was collected from country reports presented by DATAREPORTAL for January 2022, 2023, and 2024. All data was combined into five categories named Digital Penetration, Economic Accessibility of Technologies, Mobile Commerce, Device Traffic, and Operating Systems (See Fig 1). To decrease yearly fluctuations and COVID’s effect on the numbers, the arithmetic mean of the mentioned variables was calculated. Unfortunately, the author could access only 3 years of data regarding all variables. Number of countries included in the analyses is influenced by the number of countries included in the World Value Survey Cultural Map revised in 2023. Therefore, number of countries included in the analyses is 103.

To find answers to one of the mentioned research questions the author decided to apply one way Analysis of Variance (abbr. ANOVA). ANOVA is considered to be the best tool for comparing a categorical variable that consists of three or more groups with numeric ones [41]. The characteristics of analyses allow a researcher to use one-way ANOVA as the most appropriate tool. As a result, hypotheses should be stated in the following way:

H_0_: There is no statistically significant difference among group means classified by Inglehart and Welzel’s cultural map differs significantly from the overall mean of digitalization-related data.

H_1_: At least one group mean classified by Inglehart and Welzel’s cultural map differs significantly from the overall mean of digitalization-related data.

To proceed with ANOVA the author should report that requirements were fulfilled. The first assumption is focused on the normal distribution of the data [41]. Knowing that 104 countries were included in the analyses, it is correct to assume that the provided data should be normally distributed. The second assumption supports the idea that the sample consists of independent observations that are also proved to be correct. However, it is partially hard to prove the homogeneity of variances.

ANOVA assumes equal variances between the groups being compared. If the variances are not equal, it may lead to inaccurate results and conclusions [42]. If Levene’s test was applied to determine the homogeneity of variances. If the significance level is below 0.05, it means that the equal variance assumption was violated [41]. The results illustrate that data regarding Internet Users (ITU) MEAN, Active social media users MEAN, Price of the cheapest smartphone divided by average income % MEAN, and share of web traffic by Mobile OS–Android and Apple MEAN is not homogeneous.

To correct the situation with the F test, the statistical literature offers the calculation of Brown–Forsythe F [43], as well as Welch’s F [44]. The results of the variables that failed Levene’s test are illustrated below (See Table 1). The scores prove that even though the result of Homogeneity of Variances showed untrustworthy numbers, Robust Tests of Equality of Means allow us to move forward without excluding variables illustrated in Table 2.

**Table 1:** Test of homogeneity of variances.

*Dependent variables*	*Levene Statistic*	*df1*	*df2*	*Sig.*
*Cellular Mobile Connections MEAN*	1.681	7	96	0.123
*Internet Users (ITU) MEAN*	14.400	7	96	<0.001*
*Active social media users MEAN*	10.210	7	96	<0.001*
*Share of web traffic by device—Mobile phone MEAN*	0.695	7	96	0.676
*Share of web traffic by device—Laptop and Computer MEAN*	1.392	7	96	0.218
*Price of the cheapest smartphone in USD MEAN*	1.774	7	96	0.101
*Price of the cheapest smartphone in USD vs average income % MEAN*	7.298	7	96	<0.001*
*Made a purchase using mobile phone/ internet (past year) MEAN*	1.938	7	96	0.072
*Used mobile phone or internet to pay bills (past year) MEAN*	1.818	7	96	0.093
*Share of web traffic by Mobile OS—Android MEAN*	2.406	7	96	0.026*
*Share of web traffic by Mobile OS—Apple MEAN*	2.307	7	96	0.032*

Source: author’s own calculations

**Table 2:** Robust tests of equality of means.

		*Statistic^a^*	*df1*	*df2*	*Sig.*
*Internet Users (ITU) MEAN*	Welch	22.108	7	26.161	**<0.001**
	Brown-Forsythe	12.534	7	15.809	**<0.001**
*Active social media users MEAN*	Welch	19.632	7	25.875	**<0.001**
	Brown-Forsythe	14.313	7	31.901	**<0.001**
*Price of the cheapest smartphone in USD vs average income % MEAN*	Welch	12.894	7	30.496	**<0.001**
	Brown-Forsythe	15.022	7	53.392	**<0.001**
*Share of web traffic by Mobile OS–Android MEAN*	Welch	15.577	7	25.525	**<0.001**
	Brown-Forsythe	20.561	7	35.654	**<0.001**
*Share of web traffic by Mobile OS–Apple MEAN*	Welch	13.071	7	25.567	**<0.001**
	Brown-Forsythe	16.208	7	31.222	**<0.001**

a. Asymptotically F distributed.

Source: author’s own calculations

Based on the results of Robust Tests of Equality of Means it is clear that we might accept the results of one-way ANOVA as reliable. However, if the number of group members is not identical, the mentioned skew affects the accuracy of the F-test, and non-normality impacts the power of results in unexpected ways [45]. Therefore, the second research question (RQ2) covers only part of the variables that passed Levene’s test and focuses on finding the proportion of total variance determined by digitalization-related variables.

### One-way analysis of variance

Based on the results of one-way ANOVA, the null hypothesis can be easily rejected. It is evident that at least one group mean classified by Inglehart and Welzel’s cultural map differs significantly from the overall mean of digitalization-related data. The results illustrate that Cellular Mobile Connections (F(1,1) = 21.430, p < 0.001 unweighted; F(1,1) = 23.215, p < 0.001 weighted), Internet Users (F(1,1) = 8.970, p = 0.003 unweighted; F(1,1) = 24.360, p < 0.001 weighted), and Active Social Media Users (F(1,1) = 15.212, p < 0.001 unweighted; F(1,1) = 37.927, p < 0.001 weighted) all showed significant linear relationships with cultural values, indicating a strong link between higher digital engagement and culture.

The numbers for Web Traffic based on Device Type (Mobile vs. Laptop/Computer) varied, with mobile devices showing less significant association (F(1,1) = 1.043, p = 0.310 unweighted; F(1,1) = 32.181, p < 0.001 weighted) than laptops and computers (F(1,1) = 2.695, p = 0.104 unweighted; F(1,1) = 26.402, p < 0.001 weighted). Price of Smartphone divided to Average Income (F(1,1) = 5.153, p = 0.026 unweighted; F(1,1) = 23.370, p < 0.001 weighted) and Mobile OS (Android (F(1,1) = 8.653, p = 0.004 unweighted; F(1,1) = 28.646, p < 0.001 weighted) vs. Apple (F(1,1) = 14.583, p < 0.001 unweighted; F(1,1) = 31.756, p < 0.001 weighted)) both demonstrated significant associations, indicating that economic factors and technology preferences associate with cultural values. Variables related to mobile commerce activities, (i.e., Making Purchases (F(1,1) = 24.459, p < 0.001 unweighted; F(1,1) = 75.610, p < 0.001 weighted) and Paying Bills (F(1,1) = 6.617, p = 0.012 unweighted; F(1,1) = 40.150, p < 0.001 weighted) via Mobile), also exhibited and significant relationships, highlighting the impact of cultural values on digital financial services (See Table 3). Only one variable—The price of the Cheapest Smartphone showed no significant linear relationship, suggesting that this variable does not significantly impact cultural values.

**Table 3:** One way ANOVA.

				*Sum of Squares*	*df*	*Mean Square*	*F*	*Sig.*
*Cellular Mobile Connections MEAN*	Between Groups	(Combined)		31806.216	7	4543.745	6.286	<0.001
		Linear Term	Unweighted	15490.061	1	15490.061	21.430	<0.001
			Weighted	16779.961	1	16779.961	23.215	<0.001
			Deviation	15026.255	6	2504.376	3.465	0.004
*Internet Users (ITU) MEAN*	Between Groups	(Combined)		18797.928	7	2685.418	9.857	<0.001
		Linear Term	Unweighted	2443.806	1	2443.806	8.970	0.003
			Weighted	6636.446	1	6636.446	24.360	<0.001
			Deviation	12161.482	6	2026.914	7.440	<0.001
*Active social media users MEAN*	Between Groups	(Combined)		25177.303	7	3596.758	10.105	<0.001
		Linear Term	Unweighted	5414.222	1	5414.222	15.212	<0.001
			Weighted	13499.151	1	13499.151	37.927	<0.001
			Deviation	11678.152	6	1946.359	5.468	<0.001
*Share of web traffic by device—Mobile phone MEAN*	Between Groups	(Combined)		12734.788	7	1819.255	22.411	<0.001
		Linear Term	Unweighted	84.629	1	84.629	1.043	0.310
			Weighted	2612.429	1	2612.429	32.181	<0.001
			Deviation	10122.359	6	1687.060	20.782	<0.001
*Share of web traffic by device—Laptop and Computer MEAN*	Between Groups	(Combined)		10229.523	7	1461.360	17.040	<0.001
		Linear Term	Unweighted	231.093	1	231.093	2.695	0.104
			Weighted	2264.276	1	2264.276	26.402	<0.001
			Deviation	7965.247	6	1327.541	15.479	<0.001
*The price of the cheapest smartphone in USD MEAN*	Between Groups	(Combined)		50024.708	7	7146.387	1.245	0.287
		Linear Term	Unweighted	2095.008	1	2095.008	.365	0.547
			Weighted	5.076	1	5.076	.001	0.976
			Deviation	50019.632	6	8336.605	1.453	0.204
*Price of the cheapest smartphone in USD vs average income % MEAN*	Between Groups	(Combined)		19985.732	7	2855.105	8.491	<0.001
		Linear Term	Unweighted	1732.683	1	1732.683	5.153	0.026
			Weighted	7858.418	1	7858.418	23.370	<0.001
			Deviation	12127.314	6	2021.219	6.011	<0.001
*Share of web traffic by Mobile OS–Android MEAN*	Between Groups	(Combined)		19380.741	7	2768.677	22.491	<0.001
		Linear Term	Unweighted	1065.127	1	1065.127	8.653	0.004
			Weighted	3526.355	1	3526.355	28.646	<0.001
			Deviation	15854.386	6	2642.398	21.465	<0.001
*Share of web traffic by Mobile OS–Apple MEAN*	Between Groups	(Combined)		16427.333	7	2346.762	18.278	<0.001
		Linear Term	Unweighted	1872.284	1	1872.284	14.583	<0.001
			Weighted	4077.187	1	4077.187	31.756	<0.001
			Deviation	12350.145	6	2058.358	16.032	<0.001
*Made a puchase using mobile phone/ internet (past year) MEAN*	Between Groups	(Combined)		46102.553	7	6586.079	48.127	<0.001
		Linear Term	Unweighted	3347.109	1	3347.109	24.459	<0.001
			Weighted	10346.927	1	10346.927	75.610	<0.001
			Deviation	35755.626	6	5959.271	43.547	<0.001
*Used mobile phone or internet to pay a bills (past year) MEAN*	Between Groups	(Combined)		45351.383	7	6478.769	35.871	<0.001
		Linear Term	Unweighted	1195.047	1	1195.047	6.617	0.012
			Weighted	7251.739	1	7251.739	40.150	<0.001
			Deviation	38099.645	6	6349.941	35.157	<0.001

Source: author’s own calculations

The results aimed to provide the answer to Research Question 1 demonstrate substantial variations in the significance of linear relationships between different digitalization variables and cultural values. Variables related to mobile and internet use show particularly strong associations with cultural orientation, suggesting that as countries become more digitalized, shifts in cultural values are likely evident.

### General linear model—ETA square

The second research question is focused on the understanding of the theory regarding ETA Square. The General Linear Model (abbr. GLM) is a statistical framework that allows for the analysis of relationships between a dependent variable and one or more independent variables [41, 46]. This framework assumes that there is a linear relationship between the dependent variable and the independent variables. The results of one-way ANOVA analyses have already proved the mostly linear relationship.

The homogeneity of variance is an important assumption of the General Linear Model [46]. It assumes that the variance of the dependent variable is constant across all levels of the independent variables. Violating the assumption of homogeneity of variance, also known as homoscedasticity, can lead to biased parameter estimates and incorrect conclusions. To check for homogeneity of variance, researchers often use statistical tests such as Levene’s test or Bartlett’s test. Therefore, the author applied GLM for the variables that passed Levene’s test.

Partial ETA squared is a measure of effect size in the analysis of variance that indicates the proportion of variance in the dependent variable accounted for by a particular independent variable while controlling for other independent variables [41]. It is useful in assessing the strength of the relationship between the independent variable and the dependent variable, taking into account the influence of other variables in the model. In practical terms, a higher partial eta squared value signifies a stronger impact of the independent variable on the dependent variable, all else being equal. In the current work, the Eta square is a measure of effect size [47] that varies between small (from 0.1 to 0.3), medium (from 0.3 to 0.5), and high (≥0.5); it represents the proportion of variance in digitalization-related variables that can be explained by the membership based on Inglehart and Welzel’s cultural map. Based on the results of the calculations, the mean of Used mobile phone or internet to pay bills, Made a purchase using a mobile phone/ internet, and the share of web traffic by device–Laptop/Computer and Mobile phone is highly influenced by the membership based on Inglehart and Welzel’s cultural map.

Significant results were observed in the usage of mobile phones or the Internet for paying bills and making purchases (See Table 4). For bill payments, the mean square was 6324.947 with an F-value of 27.875 (p < 0.001) and a partial eta squared of 0.670, suggesting that 67.0% of the variance in this behavior is explained by cultural grouping. Similarly, for online purchases, the mean square was 6337.602, the F-value was 33.339 (p < 0.001), and the partial eta squared was 0.709, indicating that 70.9% of the behavior’s variance is related to cultural map membership.

**Table 4:** Tests of between-subjects effects.

*Independent: Inglehart and Welzel’s Cultural Map membership*						
*Dependent Variable*	*Type III Sum of Squares*	*df*	*Mean Square*	*F*	*Sig.*	*Partial Eta Squared*
*Cellular Mobile Connections MEAN*	31806.216	7	4543.745	6.286	<0.001	0.314
*The price of the cheapest smartphone in USD MEAN*	53529.172	7	7647.025	1.287	0.265	0.086
*Used mobile phone or internet to pay bills (past year) MEAN*	44274.629	7	6324.947	27.875	<0.001	0.670
*Made a purchase using mobile phone/ internet (past year) MEAN*	44363.213	7	6337.602	33.339	<0.001	0.709
*Share of web traffic by device—Laptop and Computer MEAN*	10229.523	7	1461.360	17.040	<0.001	0.554
*Share of web traffic by device—Mobile phone MEAN*	12734.788	7	1819.255	22.411	<0.001	0.620

Source: author’s own calculations

Based on the results illustrated in Table 4 which aimed to find an answer to Research Question 2, the analysis of web traffic showed significant cultural influences. For laptop and computer use, the mean square was 1461.360 with an F-value of 17.040 (p < 0.001), and a partial eta squared of 0.554, indicating that 55.4% of the variance is culturally related. Mobile phone usage for web traffic showed a mean square of 1819.255, an F-value of 22.411 (p < 0.001), and a partial eta squared of 0.620, showing that 62.0% of the variance could be explained by cultural map membership.

Cultural map membership had a substantial impact on the variance in cellular mobile connections, accounting for approximately 31.4% of the variation, underscoring the role of cultural factors in mobile technology adoption. The analysis showed a minimal influence of cultural categories on the affordability of smartphones, with only 8.6% of the variance explained by the mentioned cultural dimensions. Table 4 effectively demonstrates the significant influence of cultural factors on various aspects of digital technology use, particularly in digital transactions and web traffic patterns, with some variables showing high explanatory power as reflected in the partial eta squared values.

### Spearman’s correlation

To find an answer to Research Question 3 and determine the relationship between Traditional/Secular (TradAgg) and Survival/Self-Expression (SurvSAgg) values of the Cultural Map and digitalization-related data, the author conducted Spearman correlation analysis. Spearman correlation is a statistical technique used to measure the strength of the relationship between two variables [48]. It is particularly useful for analyzing the relationship between variables that may not have a linear relationship, as it is based on ranked data rather than actual values. The main purpose of this analysis is to determine the strength of the relationship between the variables.

All correlations are statistically significant with p-values less than 0.001, suggesting a strong reliability in the observed relationships. The strength of the relationship between TradAgg/SurvAgg and some of the digitalization-related variables (Used mobile phone or internet to pay bills; made a purchase using mobile phone/ internet and share of web traffic by mobile OS–Apple, and Internet Users) is high (correlation coefficient < 0.6) and variables move in the same direction. Laptop/Computer web traffic, Active social media users, Cellular Mobile Connections, and the price of the cheapest smartphone are significantly correlated with survival and/or traditional values; the strength of the relationship is mostly at the average level for both indicators of cultural values (between 0.5 and 0.3). Positive correlations suggest that more secular or self-expressive societies are more likely to engage with digital technologies.

Interestingly, the correlation coefficient of the Share of web traffic by Mobile OS–Android and Share of web traffic by device–Mobile phone is higher than 0.6 however, the values are negative. A negative Spearman’s correlation coefficient indicates an inverse relationship between the variables, meaning that as one variable increases, the other decreases. Negative correlations indicate that societies with more traditional or survival values engage more with certain digital technologies (i.e., Mobile phones, Android Smartphones, and price-related issues). Previous studies in the field also proved the mentioned notion [12, 37].

The results presented in Table 5 demonstrate a significant relationship between cultural values and digital engagement across societies. The strength and direction of the correlations between various digitalization variables and the cultural dimensions of Traditional/Secular and Survival/Self-Expression values provide robust evidence that cultural orientations significantly influence digital adoption and usage behaviors. Specifically, societies characterized by secular and self-expression values show greater engagement with digital technologies, as evidenced by higher usage of mobile payment systems, internet purchases, and active participation in social media. Conversely, societies with more traditional and survival-oriented values exhibit lesser engagement with these digital technologies. This pattern highlights the critical role of cultural context in the diffusion and utilization of digital technologies across the globe.

**Table 5:** Spearman’s correlation.

			*Used mobile phone or internet to pay bills (past year) MEAN*	*Made a purchase using mobile phone/ internet (past year) MEAN*	*Share of web traffic by Mobile OS–Android MEAN*	*Share of web traffic by Mobile OS–Apple MEAN*	*Share of web traffic by device–Laptop and Computer MEAN*	*Share of web traffic by device–Mobile phone MEAN*	*Active social media users MEAN*	*Internet Users (ITU) MEAN*	*Cellular Mobile Connections MEAN*	*The price of the cheapest smartphone in USD MEAN*	*Price of the cheapest smartphone in USD vs average income % MEAN*
*Spearman’s rho*	TradAgg	Correlation Coefficient	0.704**	0.755**	-0.669**	0.670**	0.587**	-0.659**	0.514**	0.664**	0.368**	0.352**	-0.509**
		Sig. (2-tailed)	<0.001	<0.001	<0.001	<0.001	<0.001	<0.001	<0.001	<0.001	<0.001	<0.001	<0.001
		N	104	104	104	104	104	104	104	104	104	104	104
	SurvSAgg	Correlation Coefficient	0.637**	0.630**	-0.617**	0.617**	0.580**	-0.635**	0.557**	0.622**	0.274**	0.309**	-0.481**
		Sig. (2-tailed)	<0.001	<0.001	<0.001	<0.001	<0.001	<0.001	<0.001	<0.001	<0.001	<0.001	<0.001
		N	104	104	104	104	104	104	104	104	104	104	104

*. Correlation is significant at the 0.05 level (2-tailed).

Source: author’s own calculations

## Discussion

The results of the calculation based on the other variables prove that mostly there is a linear relationship between digitalization-related variables and classification based on the cultural map. The mean of Active social media users, Internet Users (ITU), price of the cheapest smartphone in USD divided by average income, and share of web traffic by Mobile OS–Android/Apple showed statistically significant results that are lower than the threshold of 0.005 (See Table 3).

### Digital penetration

The global number of internet users and active social media users is increasing rapidly from 2022 to 2024, driven by expanded internet access and affordable mobile devices [8, 9]. Europe’s transition from 4G to 5G mobile connections is progressing, with 67% population penetration expected by 2025, although the adoption rate varies by region [49]. Social media use continues to rise, significantly impacting social interaction and political participation, but also raising concerns about misinformation and polarization [50].

The author found significant associations between internet users, mobile connections, and active social media users suggesting that higher levels of digital penetration facilitate shifts in cultural values. Studies such as those by Inglehart and Welzel have posited that increased access to information through digital means can lead to greater cultural shifts towards secular-rational and self-expression values, particularly in societies undergoing significant socio-economic changes [51]. There are some pieces of evidence that cultural variables impact mobile phone usage [52, 53]. Also, the study based on Hofstede’s Cultural dimensions illustrated that culture might be a predictor/moderator in the case of social media as well as new technology use [54]. Interestingly, the results of content analyses of different companies from American Turkish, and Chinese companies also proved the mentioned idea [55]. A substantial body of research suggests that social media significantly influences cultural values by promoting new forms of social interaction, personal expression, and public engagement. This influence is often seen as contributing to the shift towards more individualistic and self-expressive values, particularly among younger generations [56, 57].

### Economic accessibility of technologies

The concept of "Economic Accessibility of Technologies" is crucial, particularly in rural areas where access to technology directly influences economic outcomes. Studies have shown that initiatives aimed at bridging the digital divide can lead to increased income for participants, though these efforts also risk exacerbating economic disparities [50]. Furthermore, the use of mobile platforms in rural China illustrates how technology can economically empower users, despite initial barriers like low income and limited digital skills [1].

The lack of a meaningful relationship between the mean price of the cheapest smartphone in USD and cultural values suggests that the price without considering the economic situation influencing customers, might not be a strong indicator of cultural shifts. This could be because the absolute price does not account for the relative affordability of smartphones across different income levels and economic conditions in various countries [12]. However, when smartphone prices are considered relative to the average income in the country, a significant relationship with cultural values emerges. The notion indicates that the economic burden or affordability of smartphones, (i.e., rather than their absolute cost) aligns more closely with cultural characteristics defined by Inglehart and Welzel. Such a finding supports the idea that economic accessibility and the proportion of disposable income spent on technology can be a more relevant measure of how technological adoption influences cultural values.

In the case of handsets’ price, there is some evidence that culture might indirectly impact the choice of mobile phone [58]. However, it was hard to find a direct link supporting the statistical difference of group mean classified by Inglehart and Welzel’s cultural map and the price of the cheapest smartphone divided by average income. Research often discusses how the affordability of digital technology influences its adoption and subsequent cultural impact [59]. It is generally assumed that more affordable technology leads to wider access and deeper social integration [1, 50]. Still, there is an indication regarding the fact that the placement of countries in the cultural map is somewhat associated with the economic development level [60]. This notion might be supported by results of the price of the cheapest smartphone divided by average income, and share of web traffic by Android and iOS operating systems.

### Mobile commerce

The strong and significant correlations found between activities like making purchases and paying bills via mobile phones and cultural values align with these studies. This suggests that as people engage more with mobile commerce, there are noticeable shifts towards values emphasizing self-expression and modernization, reflecting a broader cultural adaptation to digital lifestyles.

The findings indicate a shift towards more secular and self-expressive values, facilitated by the ease and ubiquity of mobile financial interactions. Literature suggests a strong correlation between the adoption of mobile commerce and shifts in consumer behavior and values, especially in developing economies [61] where mobile technology leapfrogs more traditional forms of commerce [62]. Some studies [63, 64] highlight that Taobao Villages serve as a prime example of digitalization. These rural communities in China, where residents operate online businesses via Alibaba’s Taobao platform, have transformed into e-commerce hubs, driving local economic growth by facilitating online sales and job creation. In the above-mentioned case, mobile commerce can also be a driver of cultural change, promoting values such as innovation and pragmatism. It often introduces new social norms and behaviors, which can be particularly impactful in regions undergoing rapid digital transformation [65].

### Device traffic

The surge in data traffic decreased significantly due to the COVID-19 pandemic in 2021. While the pandemic is intensifying the need for universal coverage, the reduction in capital expenditure in developing countries has exacerbated the digital divide [66]. However, it is expected to fully normalize and return to an annual increase of 20–25% once the effects of the pandemic have completely subsided [49]. The findings, showing stronger associations for mobile web traffic with cultural values compared to laptop and computer traffic, align with the suggestion that mobile devices play a more crucial role in integrating and reflecting current cultural trends. Studies [67, 68] have examined how different devices (e.g., mobiles vs. laptops) mediate cultural consumption and participation differently. Mobile devices, often used on the go, are linked with more immediate and personal cultural interactions compared to more stationary uses like laptops [67]. Also, mobile devices are often linked with higher levels of engagement in cultural activities, particularly social media, and online communities, which can foster greater exposure to diverse cultural norms and global interactions [68]. However, laptops and desktop computers might still be preferred for educational or professional tasks and could therefore be associated with different sets of cultural values compared to mobile phones [69].

### Operating systems

A significant relationship was found with different mobile operating systems underlining the role of technology choice as a reflection of broader cultural and social patterns. These findings suggest that operating system preferences are not merely about technology but also reflect broader socio-economic [12] and cultural values [70]. The specific operating system (Android vs. Apple) preference has been linked to different socio-economic demographics and cultural preferences [71]. These preferences can reflect deeper cultural orientations and identities. The significant correlations found with different mobile operating systems underline the role of technology choice as a reflection of broader cultural and social patterns.

In conclusion, the current results largely support the existing literature on the transformative role of cultural values in shaping digitalization, with nuanced differences in how economic factors like affordability interact with cultural change. This comparison highlights the dynamic interplay between technology adoption and cultural evolution.

## Conclusion

The analyses emphasize the critical impact of cultural values on digitalization, revealing that cultural values impact the behavioral patterns towards the use of some products (i.e., laptop, smartphone), applications (social media, mobile payment, mobile purchase, etc.), and services (internet, mobile phone number, etc.). By leveraging data from the Inglehart-Welzel Cultural Map for comparison with digitalization-related variables, this study sheds new light on their interconnection and enhances our understanding of the digital divide, which corresponds with JEL codes O33 and O14.

Specifically, the statistical analyses conducted to address Research Question 1 indicate that at least one group mean, classified by Inglehart and Welzel’s cultural map, significantly diverges from the overall mean of digitalization-related data. A closer examination of web traffic by device type illustrates that while mobile devices show a less pronounced association with user behavior compared to laptops and computers, they still play a role in shaping cultural consumption patterns. Significant relationships were found between digital penetration as well as economic accessibility and cultural values, particularly regarding smartphone prices relative to average income and mobile operating systems (Android and Apple). Additionally, mobile commerce activities, such as making purchases and paying bills, demonstrated significant association with cultural values, suggesting that country classification based on the values influences the use of digital financial services. The price of the cheapest smartphone (in USD) did not show a significant relationship, which might be related to the numeric characteristic of the variable nevertheless the mean of the price of the cheapest smartphone divided by average income is statistically different from the mean of the cultural values.

The statistical analyses conducted to address Research Question 2 indicate that 70.9% of the variance in purchases made online or by mobile phones and 67.0% of the variance in bills paid online or by mobile phones is related to cultural map membership, suggesting that the values and norms inherent in different cultures influence consumers’ online shopping habits. Furthermore, 62.0% of the variance in mobile phone web traffic and 55.4% in laptop and computer web traffic is linked to cultural map membership, highlighting how cultural values impact technology adoption in these device categories. Moreover, it was observed that cultural map membership accounts for approximately 31.4% of the variation in cellular mobile connections, demonstrating that cultural factors significantly impact the adoption of mobile technology across different societies. However, despite its overall influence, the impact of cultural dimensions on smartphone affordability is relatively minimal, explaining only 8.6% of the variance, thus indicating that other factors also play a crucial role in determining smartphone accessibility.

The statistical analyses carried out to find an answer to Research Question 3 proved that cultural values defined by Inglehart and Welzel’s cultural map, have a significant impact on digitalization variables such as mobile connectivity, digital transactions, and web traffic by device (Spearman’s Correlation). Especially, variables like using a mobile phone or the internet to pay bills, making a purchase using a mobile phone/ internet, the iPhone’s share in web traffic, and the percentage of Internet Users are strongly influenced by cultural values, explaining over 60% of the variance in these areas. These findings underscore the importance of considering cultural values when implementing policies and strategies aimed at enhancing digital inclusion and technological adoption across different societies. However, the strength of the relationship between Android’s share of web traffic, and the share of mobile web traffic also exceeds 0.6, yet the values remain negative. It is closely related to the fact that users with higher scores of survival and/or traditional values, based on their economic conditions, tend to mostly use inexpensive Android mobile phones. This notion is further supported by the average strength of the negative relationship between the price of the cheapest smartphone in USD and traditional/survival values. Laptop/Computer web traffic, Active social media users, Cellular Mobile Connections, and the price of the cheapest smartphone are significantly correlated with survival and/or traditional values; the strength of the relationship is mostly at the average level. These findings based on Spearman’s Correlation indicate that higher engagement with the internet, mobile connections, and social media strongly correlates with shifts toward more secular and self-expressive values. Additionally, the use of mobile commerce and the choice of operating systems reflect deeper socio-economic and cultural trends, with mobile devices and different operating systems (Android vs. iOS) aligning with specific cultural orientations.
